# Short-Term Surgical Outcomes for Lobectomy Between Robot-Assisted Thoracic Surgery and Uniportal Video-Assisted Thoracoscopic Surgery

**DOI:** 10.3389/fonc.2022.914059

**Published:** 2022-07-13

**Authors:** Fan Zhang, Lin Xu, Hongda Lu, Anqun Ma, Gongchao Wang

**Affiliations:** ^1^ Department of Thoracic Surgery, Shandong Provincial Hospital Affiliated to Shandong First Medical University, Jinan, China; ^2^ School of Nursing and Rehabilitation, Shandong University, Jinan, China

**Keywords:** RATS, UVATS, lung cancer, lymph node dissection, short-term outcomes

## Abstract

**Objectives:**

To evaluate the short-term outcomes of uniportal video–assisted thoracoscopic surgery (UVATS) and Da Vinci robot–assisted thoracoscopic surgery (RATS) in lobectomy and lymph node (LN) dissection.

**Methods:**

The two groups of patients with primary non-small cell lung cancer (NSCLC; RATS group, UVATS group) were matched by the propensity score to compare LN dissection and recent clinical outcomes. The results were analyzed by univariate analysis. From November 2020 to November 2021, 412 NSCLC patients (54 RATS and 358 UVATS) from a single institution of the Provincial Hospital affiliated with Shandong First Medical University were included in the analysis. Age, sex, lung lobe, surgical resection scope, solid nodules, and core tumor ratios were matched according to different surgical methods.

**Results:**

From November 2020 to November 2021, 412 patients with NSCLC (54 RATS, 358 UVATS) from the Provincial Hospital affiliated with Shandong First Medical University were included in the analysis. According to our matching results, LN dissection was more thorough in the RATS group.

**Conclusion:**

RATS has potential advantages over UVATS in radical lung cancer surgery.

## Introduction

The evolution of technology has gradually promoted the development of minimally invasive surgery, and the prospect of minimally invasive surgery for non-small cell lung cancer (NSCLC) has changed dramatically. Robot-assisted thoracoscopic surgery (RATS) and video-assisted thoracoscopic surgery (VATS) are less-invasive methods for radical lung cancer surgery (1).The minimally invasive surgery provides a better postoperative quality of life, reduced complications, and less length of hospital stay than open-heart surgery (2). After uniportal thoracoscopic surgery was first used for a wedge resection of the lung (3), more and more thoracic surgeons developed the uniportal thoracoscopic technique. Multiple studies have shown that uniportal video–assisted thoracoscopic surgery (UVATS) incision can shorten the operation time and reduce long-term postoperative pain (1, 4). In 2011, an article described the potential of Da Vinci robotic–assisted thoracoscopy in surgery (5), and a small number of surgeons applied robotic surgery to treat lung cancer.

Currently, a large amount of data support the feasibility, safety, and effectiveness of minimally invasive techniques. In recent years, UVATS and RATS have increased in number and proportion in minimally invasive areas. However, a recent analysis showed that the total number of lymph nodes (LNs) resected by VATS was small. The Da Vinci surgical system (DVSS) offers the benefits of joint forceps, including the three-dimensional (3D) free field of vision, these can improve the accuracy and quality of LNs (6, 7). The composition of pulmonary nodules has not been paid much attention before, so few reports compare pulmonary nodules with different core tumor ratios (CTRs) in RATS and UVATS.

Previously an academic thoracic surgery center with VATS for minimally invasive anatomic pulmonary resection, we now added the RATS program. This study aimed to analyze the cases of patients receiving RATS and UVATS during the same period of the continuous treatment of stage I–IIIA primary NSCLC in our hospital, which compare the short-term efficacy of the two surgical methods in our institution.

## Materials and Methods

### Patient Selection

The Ethics Review Committee approved the study of the Provincial Hospital affiliated with Shandong First Medical University. The data came from 412 patients who underwent lung cancer surgery at the facility in November 2020 and November 2021.

Inclusion criteria included the following: 1. preoperative pulmonary function supported lobectomy, preoperative computed tomography (CT) showed non-pure ground glass density nodules, and there was only one surgical method; 2. pathologically confirmed stage I–IIIA NSCLC, requiring LN dissection; 3. preoperative radiotherapy, chemotherapy, puncture, pulmonary nodule ablation, and other treatments were not performed; and 4. did not undergo any lung surgery. Patients who met the criteria were enrolled in the study.

Excluded criteria included the following: 1. the lung has undergone surgery; 2. extensive adhesion and atresia in the pleural cavity; and 3. intraoperative exploration revealed tumor-infiltrating surrounding organs and invading the pleura, requiring the simultaneous removal of a lung and other thoracic organs. Operative death was defined as death within 30 days of the operation or any time after the operation if the patient did not leave the hospital alive.

**Figure 1:**
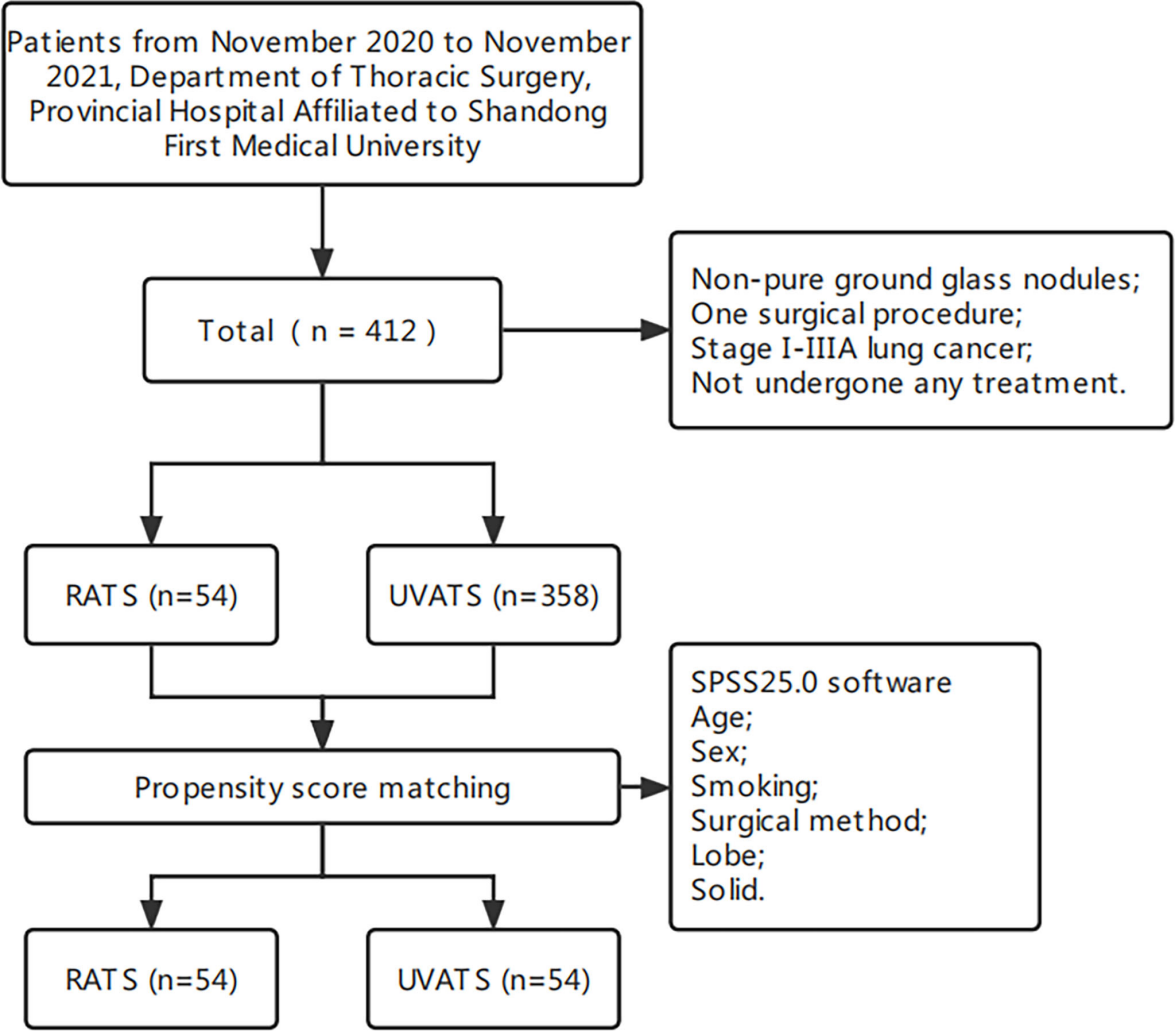
Flow-chart of the study.

The choice of surgical method depends on the patient’s will. Patients were retrospectively classified into two groups based on the surgical approach: RATS and UVATS. We made a short flowchart, as shown in **Figure 1**.

### Surgical Technique

Preoperative patients undergoing surgery at our center have met the surgical standards recommended by the NCCN guidelines (8) and have undergone a multidisciplinary consultation with physicians in the departments of oncology, thoracic surgery, and respiratory medicine before hospitalization. We have considered the choice of tumor treatment and performed the surgery.

Patients in the RVTS group were in a lateral decubitus position. One surgical incision and three robotic arm incisions were opened while maintaining a distance of 10 cm between each port and 10–15 cm from the operating site; the camera is on the middle port. Patients in the VATS group were in a lateral decubitus position. According to the surgeon’s preference, a surgical incision was opened in the 4th or 5th intercostal space. The camera was placed on the side of the incision away from the surgeon and secured by an assistant to expose the field of vision.

All patients received routine preoperative examination and serological examination in our hospital, and several physicians decided the preoperative surgical plan through discussion. General anesthesia was used for surgery, and a one-lung ventilation and incision protector was placed in all incisions. Energy equipment was used to anatomize the lung structure. According to the recommendations of the NCCN guidelines, patients with resectable NSCLC should receive N1 and N2 nodule resection and at least 3 N2 station sampling or LN dissection, including 2, 4, 7, 8, and 9 stations on the right and 5, 6, 7, 8, and 9 on the left (8).

We formulated the extubation conditions by clinical specifications and extubation strategy based on clinical experience: 1. the absence of air leakage;2. the absence of an increased drainage volume every 6 h after surgery;3. the absence of a densely bloody, purulent, or cloudy pleural effusion;4. the absence of atelectasis on postoperative chest radiograph; and 5. the absence of subcutaneous emphysema. Patients meeting the above conditions and having less than 200ml of drainage per day were removed.

### Study Variables

We obtained age, sex, procedure, surgical location, and smoking history from medical records. We got the patient’s height, weight, postoperative daily drainage volume, and pain score on the first day after surgery from the nursing record paper. Postoperative thoracic drainage volumes were calculated. The characteristics of the target nodules, including solid nodules, subsolid nodules, and ground-glass nodules, were obtained from the imaging reports. According to the Visual Analog Scale for Pain, postoperative pain was scored. CTR is the ratio of solid core-to-length diameter on the maximum tumor section in preoperative CT imaging. TNM staging is based on the Joint Committee on Cancer Staging Manual (8th Edition) (9).

Differences in the characteristics of patients in the surgical group suggest that treatment allocation is affected by selection bias. Therefore, we built the propensity score matching model. Each patient receiving VATS was matched with one RATS (probability <2%) to form a surgical group with a similar probability of being assigned to each surgical type. Propensity score–matched variables are presented in the results, and the objective partially eliminates the bias that usually accompanies treatment assignment in non-randomized studies.

### Statistical Analysis

We analyzed all the patients’ factors. The continuous variables are summarized as the mean ± standard deviation of normally distributed data and the median [interquartile range (IQR)] of non-normally distributed data. For categorical variables, the Mann–Whitney U test was performed for a comparison between the two groups. All statistical tests were two-tailed tests, and P < 0.05 was considered statistically significant. SPSS25.0 software was used for propensity score matching. The graphics were created with the help of GraphPad Prism.

## Results

### Patient Characteristics in the Unmatched Cohort

A total of 412 patients were collected, and the clinical characteristics of the collected patients were described at baseline according to different surgical methods, as shown in **Table 1**. The patients were divided into two groups according to surgical methods, including 54 RATS and 358 UVATS patients. The median age was 57 years; male patients accounted for 39.3% (n=162). Smoking history accounted for 16% (n=66). Lobectomy accounted for 97.1% (n=400). Patients with stage pI tumor accounted for 93% (n=383). Solid nodules accounted for 90% (n=371). CTR > 0. 5 accounted for 69.2% (n=285). The median postoperative hospital stay was 3 days. The median number of days with a chest tube was 2 days. The median pleural drainage volume was 280 ml. Lung air leakage occurred in 11.9% of patients after surgery (n=49). No perioperative death or open-chest surgery occurred in all patients during the observation period. These results can be seen in **Table 1**.

**Table 1:** Patient characteristics in the unmatched cohort (N = 412).

Characteristics	Total	RATS (n = 54)	VATS (n = 358)	P
**Age (year, IQR)**	57 (50–64)	61 (53–67)	57 (49–64)	0.015
**Sex male (n, %)**	162 (39.3)	23 (42.6)	139 (38.8)	0.598
**Lobe (n, %)**				0.268
**RUL**	147 (35.7)	20 (37.0)	127 (35.5)	
**LUL**	87 (21.1)	16 (29.6)	71 (19.8)	
**RML**	29 (7)	4 (7.4)	25 (7)	
**RLL**	66 (16.0)	6 (11.1)	60 (16.8)	
**LLL**	83 (20.1)	8 (14.8)	75 (20.9)	
**Smoking (n, %)**				0.592
**Never**	346 (84.0)	44 (81.5)	302 (84.4)	
**Former**	66 (16.0)	10 (18.5)	56 (15.6)	
**Operation method (n, %)**				0.173
**Pulmonary segments**	12 (2.9)	0 (0)	12 (3.4)	
**Pulmonary lobectomy**	400 (97.1)	54 (100)	346 (96.6)	
**Pathology (n, %)**				0.164
**Adenocarcinoma**	387 (93.9)	53 (98.1)	334 (93.3)	
**Squamous**	25 (6.1)	1 (1.9)	24 (6.7)	
**pT stage (n, %)**				0.023
**1a**	158 (38.3)	13 (24.1)	145 (40.5)	
**1b**	158 (38.3)	25 (46.3)	133 (37.2)	
**1c**	67 (16.3)	10 (18.5)	57 (15.9)	
**2a**	24 (5.8)	3 (5.6)	21 (5.9)	
**2b**	2 (0.5)	1 (1.9)	1 (0.3)	
**3**	3 (0.7)	2 (3.7)	1 (0.3)	
**pN stage (n, %)**				0.091
**N0**	382 (92.7)	47 (87.0)	335 (93.6)	
**N1**	14 (3.4)	4 (7.4)	10 (2.8)	
**N2**	16 (3.9)	3 (5.6)	13 (3.6)	
**Solid (n, %)**	371 (90)	51 (94.4)	320 (89.4)	0.248
**CTR (n, %)**				0.075
**≤0.5**	127 (30.8)	11 (20.4)	116 (32.4)	
**>0.5**	285 (69.2)	43 (79.6)	242 (67.6)	
**Length of tumor (cm, IQR)**	13.5 (10–20)	15 (10.8–25)	13 (10–20)	0.053

RUL, right upper lobe; LUL, left upper lobe; RML, right middle lobe; RLL right lower lobe; LLL, left lower lobe; CTR, core tumor ratio.

### Patient Characteristics of the Propensity Score–Matched Patients

According to surgical methods, using propensity score matching, all patients’ data were matched with SPSS software, and the primary data were age, sex, smoking, operation method, lobe, and solid. In the end, 108 patients were obtained, and the clinical baseline characteristics after matching are shown in **Table 2**. They had similar clinical features.

**Table 2:** Patient and disease characteristics of the propensity score–matched groups (N = 108).

Characteristics	RATS (N= 54)	VATS (N= 54)	P
**Age (year, IQR)**	61 (53–67)	60 (51–65)	0.449
**Sex Male (n,%)**	23 (42.6)	21 (38.9)	0.697
**Lobe (n,%)**			0.251
**RUL**	20 (37.0)	23 (42.6)	
**LUL**	16 (29.6)	20 (37.0)	
**RML**	4 (7.4)	2 (3.7)	
**RLL**	6 (11.1)	5 (9.3)	
**LLL**	8 (14.8)	4 (7.4)	
**Smoking (n,%)**			0.809
**Never**	44 (81.5)	43 (79.6)	
**Former**	10 (18.5)	11 (20.4)	
**Operation method (n,%)**			1.000
**Pulmonary segments**	0 (0)	0 (0)	
**Pulmonary lobectomy**	54 (100)	54 (100)	
**Pathology (n,%)**			0.028
**Adenocarcinoma**	53 (98.1)	47 (87.0)	
**Squamous**	1 (1.9)	7 (13.0)	
**pT stage (n,%)**			0.509
**1a**	13 (24.1)	16 (29.6)	
**1b**	25 (46.3)	24 (44.4)	
**1c**	10 (18.5)	8 (14.8)	
**2a**	3 (5.6)	6 (11.1)	
**2b**	1 (1.9)	0 (0)	
**3**	2 (3.7)	0 (0)	
**pN stage (n,%)**			0.811
**N0**	47 (87.0)	48 (88.9)	
**N1**	4 (7.4)	2 (3.7)	
**N2**	3 (5.6)	4(7.4)	
**Solid (n,%)**	51 (94.4)	52 (96.3)	0.649
**CTR (n,%)**			0.809
**≤0.5**	11 (20.4)	10 (18.5)	
**>0.5**	43 (79.6)	44 (81.5)	
**Length of tumor (cm, IQR)**	15 (10.8-25)	15 (10-22)	0.587

RUL, right upper lobe; LUL, left upper lobe; RML, right middle lobe; RLL, right lower lobe; LLL, left lower lobe; CTR, core tumor ratio.

**Figure 2:**
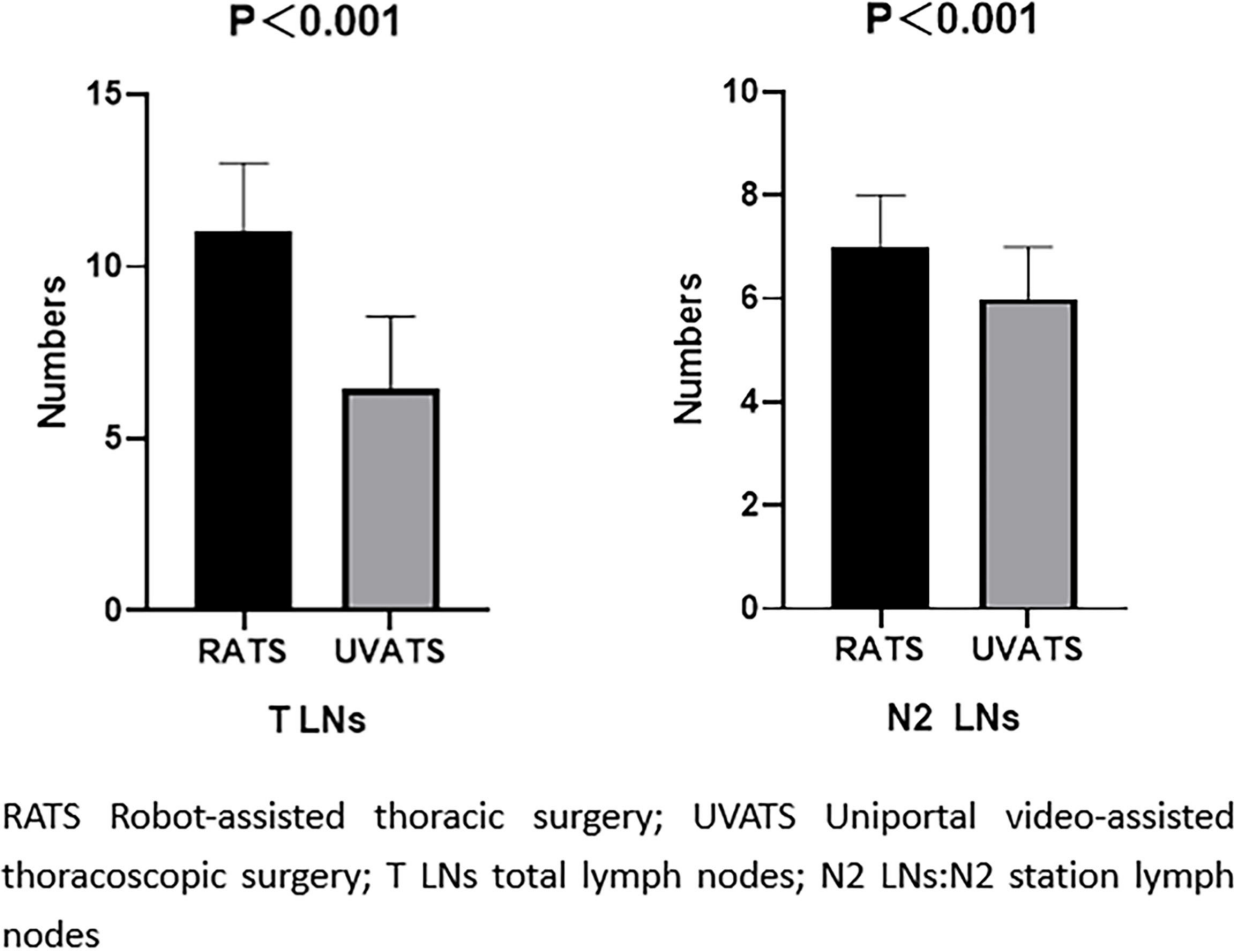
Comparison of the number of lymph node dissection in matched cohort. The model was adjusted for age, sex, smoking, lobe, operation method, solid.

Matched-cohort RATS had an advantage over UVATS in the number of LN dissections (**Figure 2**). In both groups, the most common postoperative complication is lung leakage. In the VATS group, one patient was recatheterized due to extensive subcutaneous emphysema. The other patient accidentally pulled out the chest tube while going to the toilet, and there were no apparent complications when he was discharged. There was no statistical difference in the postoperative pulmonary air leakage incidence between the two groups (P=0.223). There was no significant difference in pain on the first postoperative day (P=0. 055), but the mean length of stay at UVATS was shorter (P<0.001). The median number of mediastinal LN dissection and the total number of LNs obtained by RATS were higher than those by UVATS (P<0. 001 for both factors). The cost of surgery in the RATS group was higher than that in the UVATS group (P<0. 001). **Table 3** shows the statistics of the short-term outcome of the matched population.

**Table 3:** Postoperative outcomes of the propensity score–matched groups (N =108).

Characteristics	RATS (N= 54)	VATS (N= 54)	P
**T LNs (n, IQR)**	11 (10–13)	6 (5–7)	**<0.001**
**N2 LNs (n, IQR)**	7 (6–8)	6 (5–7)	**<0.001**
**Air leakage (n,%)**	4 (7.4)	8 (14.8)	0.223
**LOS (day, IQR)**	4 (3–5)	3 (2–3)	**<0.001**
**Drainage time (d, IQR)**	2 (2–3)	2 (1–2)	**0.001**
**PDV (ml, IQR)**	475 (320–757.5)	255 (160–382.5)	**<0.001**
**Cost (CNY, IQR)**	74,998.5 (65,473.5–75,486.6)	45,180.6 (35,833.1–54,869.4)	**<0.001**
**Pain (score, range)**	2 (1–4)	2 (1–4)	0.055

TLNs, total lymph nodes; N2 LNs, N2 station lymph nodes; LOS, length of hospital stay; PDV, pleural drainage volume.

## Discussion

According to the GLOBOCAN (Global Cancer) statistics, there were approximately 1 million cases of lung cancer worldwide in 2000 and an estimated 2.09 million new cases in 2018 (10). Surgery is the primary treatment for lung cancer, especially NSCLC. In the initial thoracoscopic surgery, there are more than two surgical ports. Thoracic surgeons have been pursuing the innovation of surgical methods. RATS and UVATS have been widely used in treating lung cancer, and the NCCN guidelines have designated them as the first choice for radical lung cancer surgery. At present, the prospect of minimally invasive surgery in lung cancer treatment has changed dramatically. However, LN dissection plays a vital role in the radical resection of lung cancer, which can clarify postoperative staging, guide postoperative adjuvant therapy, and prolong the disease-free survival time. The quality of LN dissection, including the number of LNs dissected, is an indirect indicator of the surgical thoroughness of lung cancer (11).

In this study, no patients were transferred to thoracotomy or died. Before matching, the pulmonary air leakage complication rate was 7.4% in the RATS group and 12.6% in the UVATS group. After being matched, there was no significant difference in postoperative complications between the two groups. We found a statistical difference in the number of LN dissections between the two groups in postoperative observation. The number of LN dissection in the RATS group was significantly higher than that in the UVATS group (the median value of RATS was 11, and the median value of UVATS was 16; P < 0.001). Although some previous prospective studies have shown that RATS and VATS can achieve the same tumor outcome, there is no difference in LN dissection between the two surgical approaches. However, recent studies have shown that RATS can remove more LNs and obtain more positive LNs (2, 7).

This study suggested that the total number of dissected LNs in the mediastinal region of RATS was significantly higher than that of the UVATS group. Our study finding is similar to recent studies that RATS have a more significant advantage than VATS in LN dissection at the N2 station (6, 12). In a large retrospective study of 7,452 matched stage I lung cancer patients, the comparison results also suggested that the median number of LNs dissected by robotic surgery was higher than thoracotomy (13). Yang et al. (14) also suggested that RATS has certain advantages over UVATS in treating lung cancer and LN dissection in small-sample-size studies. In contrast, UVATS is often accompanied by a mutual interference of instruments due to the limitation of the fixed-angle field of vision, which makes it challenging to perform LN dissection with UVATS. In this study and similar to our results, we analyze why more LNs may be that the robot surgery has better operative field exposure in the intraoperative, flexible mechanical arm, more thorough cleaning of LNs, and more accurate operation to the mediastinum and hilar LNs in the deeper position.

In terms of postoperative recovery, in this study, we found that the RATS group had more postoperative pleural drainage volume, drainage time, and postoperative hospital stay than the UVATS group. Drainage tube placement is routinely required in chest surgery patients, and the extubation time is closely related to postoperative drainage. The increased pleural drainage volume in the RATS group is as follows: RATS can obtain more LNs in the mediastinal area and destroy more mediastinal regions. RATS has four surgical incisions, which destroy more parietal pleura and affect pleural drainage fluid reabsorption to a certain extent. Some studies have found that age is an independent risk factor for increased total pleural drainage. Lower pneumonectomy is also a factor in increased pleural drainage (15).

Although not all postoperative patients were systematically assessed for pain scores in this study, there were no significant differences in early postoperative pain in lung cancer patients. This result is similar to the study of Van der Ploeg APT (16). We speculate that compared with thoracotomy, the smaller surgical incision in minimally invasive surgery reduces the injury of the intercostal nerve, thus reducing postoperative pain. Compared with traditional thoracotomy, the small incision of RATS and UVATS surgeries did not have the expansion of an intercostal space. Minimally invasive surgery can significantly shorten the operation time, to a certain extent, reduce the compression and damage time to the intercostal nerve, and reduce postoperative pain. However, this study did not systematically evaluate patients’ pain. Currently, our study lacks comparative studies on long-term postoperative pain in patients with UVATS, and more randomized trials are needed to confirm this in the future.

In the study, the hospitalization cost of the robot-assisted thoracoscopic surgery group was significantly higher than that of the UVATS group, which is also one of the problems that robotic surgery faces. Although the benefits of robot-assisted surgery are apparent, RATS is more expensive than other methods. The price of robotic surgical systems and their corresponding surgical instruments is high because technology monopolizes production in this field. Novellis et al. (17) reported that the Da Vinci surgical system costs approximately US$200,000 per year to maintain and $2 million to produce expensive one-off consumable items. Hospital costs will eventually be transferred to patients through higher insurance premiums, which naturally make surgery expensive. Moreover, this part of the cost is not covered by medical insurance, and patients have to bear it themselves, which makes it difficult for the Da Vinci surgical system to be widely used. Rising health spending can be a real problem.

UVATS has become the most exciting new development in minimally invasive thoracic surgery. While ensuring safety and oncology results, the single 4–5-cm surgical approach minimizes surgical trauma, alleviates postoperative pain, and contributes to rapid postoperative recovery (15). The DVSS combines surgical safety with a 3D imaging system, a mechanical arm that can ignore hand tremors, and action lever reduction technology to perform delicate soft tissue dissection (18, 19). RATS can also be applied to surgical cases with more complex anatomy, such as obese patients and after neoadjuvant therapy. The unique advantages of the two surgical methods make them widely used in the treatment of lung cancer in thoracic surgery.

### Limitation

There were some limitations in our study. Although we had more cases of UVATS, the number of RATS studied was very small. In addition, our study was limited to our institution and was a single-center study. Our study was done recently, and we did not predict long-term survival. Focusing only on a specific procedure can lead to different results than in previous studies by such bias in studies.

## Conclusion

For stage I–IIIA NSCLC with solid nodules, in our study, LN dissection can benefit from RATS, which can perform better anatomy and has potential benefits for the postoperative tumor staging of patients.

## Data Availability Statement

The raw data supporting the conclusions of this article will be made available by the authors, without undue reservation.

## Ethics Statement

The studies involving human participants were reviewed and approved by Medical Ethics Commission of Shandong Provincial Hospital SWYX: No. 2022-261. Written informed consent for participation was not required for this study in accordance with the national legislation and the institutional requirements.

## Author Contributions

FZ and GW designed the study and wrote the manuscript. HL and AM are responsible for data collection. LX and GW revised the manuscript and finally approved the submitted and published versions. GW is the guarantor of this work and, as such, had full access to all the data in the study and takes responsibility for the integrity of the data and the accuracy of the data analysis. All authors contributed to the article and approved the submitted version.

## Funding

This work was supported by the Natural Science Foundation of Shandong Province ZR2015HM066.

## Conflict of Interest

The authors declare that the research was conducted in the absence of any commercial or financial relationships that could be construed as a potential conflict of interest.

## Publisher’s Note

All claims expressed in this article are solely those of the authors and do not necessarily represent those of their affiliated organizations, or those of the publisher, the editors and the reviewers. Any product that may be evaluated in this article, or claim that may be made by its manufacturer, is not guaranteed or endorsed by the publisher.
